# The Impact of Strabismus Surgery on Irish Adults

**DOI:** 10.22599/bioj.107

**Published:** 2018-04-24

**Authors:** Barry Power, Melissa Murphy, John Stokes

**Affiliations:** 1 Mater Misericordiae University Hospital, IE; 2 Galway University Hospital, IE; 3 University Hospital Waterford, IE

**Keywords:** Adult Strabismus, Strabismus Surgery, Quality of Life, Strabismus, AS-20

## Abstract

**Aims:**

Our primary objective was to evaluate our adult strabismus service and the impact strabismus surgery has on quality of life (QOL) in patients from an Irish cohort. Our secondary objective was to compare QOL outcomes across different subgroups.

**Methods:**

A service evaluation was prospectively performed over an 18-month period. We prospectively audited the preoperative and postoperative QOL scores from 35 adult strabismus procedures using the adult strabismus score (AS-20) (0–100).

**Results:**

Postoperative patients achieved an average 14.22 score increase in QOL (p = 0.0018). Females showed lower preoperative scores (46.78 vs. 60.89; p = 0.047) and a trend towards larger increases compared to males (21.05 vs. 51.12; p = 0.1). No significant difference was detected between primary and recurrent strabismus repairs (18.10 vs. 16.55; p = 0.4). Lower preoperative scores (0–33) were associated with higher increases compared with moderate (34–66) and high (67–100) preoperative scores (33.47, 12.03, –4.57 respectively). Patients reporting QOL score decreases after surgery were more likely to come from the high preoperative score group than the moderate or low groups (50%, 19% and 22% respectively).

**Conclusion:**

We demonstrate that strabismus surgery has a significant positive impact on QOL scores in Irish adults. We show that patients with high preoperative QOL scores may have a greater chance of QOL score decreases postoperatively, despite good clinical alignment. We believe greater preoperative discussion around patient expectations in these cases, may improve subjective postoperative results.

## Introduction

Adult strabismus affects approximately 4% of the population (Martinez-Thompson et al. 2014). It can cause significant functional visual problems including diplopia, impaired depth perception and loss of binocular single vision. However, in many cases it is the psychosocial impact that causes the most distress to patients. Adult strabismus has proven links with mental illness, with the prevalence of mental illness found to be 10% higher than age-matched controls in a study of 297 adult strabismus patients (Hassan, Hodge and Mohney 2015). Adults with strabismus report higher rates of low self-esteem, social anxiety and difficulty with interpersonal relationships than the general population (Burke, Leach and Davis 1997; Jackson et al. 2006; Satterfield 1993). This anxiety can have a profound effect on social lives and careers.

As well as the personal impact strabismus has, it is also reported to be associated with conscious and subconscious prejudice from others. A Swiss study asked 40 recruitment consultants to rate candidates employability based on photographs. The study found that 72.5% of recruiters were less likely to hire patients with strabismus. Candidates showing an ocular deviation were rated less intelligent and attractive (Mojon-Azzi and Mojon 2009). These social prejudices have been shown to start in childhood. In another study, children were asked to invite other children to an imaginary birthday party based on photographs. Some of the images were digitally altered to display strabismus. From the age of 6, the selectors showed a negative bias against children with strabismus. This bias progressively increased with age (Mojon-Azzi, Kunz and Mojon 2010).

Researchers have compared strabismus to other conditions using mean utility. This method asks patients to estimate their own life expectancy and asks them how many years they would give away to be completely rid of a condition and all of its effects. The formula is 1-years traded/life expectancy. A score of 1 means no time traded and the lower the score the more time traded. Preoperative strabismus scores 0.85, similar to mild diabetic retinopathy (0.83) and to a mild cerebrovascular incident (0.85) or partially controlled epilepsy (<2 seizure per month) (0.91) (Bell et al. 2001). In terms of quality-adjusted life years (QALYs), strabismus surgery resulted in a mean cost-utility of $1,632/QALY. In the United States, treatments costing less than $50,000/QALY are considered “very cost-effective” (Beauchamp et al. 2006).

In recent times, there has been an increased drive amongst some healthcare providers and governments to designate strabismus surgery as cosmetic. This label effectively removes strabismus surgery as a publicly funded procedure and potentially impacts on insurance policy coverage. It also undervalues a procedure with the potential for huge patient quality of life (QOL) improvement. It is not appropriate to label a procedure restoring normality, from what is a pathologic change, as cosmetic. Our aim was to demonstrate the efficacy of adult strabismus surgery in an Irish population utilising a QOL assessment measurement tool.

## Methods

This prospective service evaluation analysed 35 patients listed for adult strabismus surgery over an 18-month period in an Irish university hospital. This audit and data collection conformed to all local laws and was compliant with the principles of the Declaration of Helsinki. We enrolled all consenting adult patients listed for surgery. Patients were asked to fill out a pre-operative questionnaire after they were listed for their operation and were to fill out the same questionnaire a minimum of six weeks postoperatively. The mean follow up time was 11 weeks postoperatively (range of 6–24).

We included cases of primary and recurrent strabismus. In using the term recurrent strabismus, we refer to patients that developed strabismus years after a successful initial operation, usually in childhood or adolescence. We excluded two complex cases of adult strabismus in which surgery was undertaken with the knowledge that ocular alignment was unlikely with one operation alone. These patients did not fill out postoperative questionnaires as further surgery was planned. A single consultant surgeon performed all procedures. Patients were consented to allow us to use their anonymised data in the analysis and no patients declined to be included. Those under the age of 18 or unable to consent were not included.

The validated adult strabismus (AS-20) questionnaire was used to evaluate the quality of life (Hatt et al. 2009). The questionnaire features a psychosocial and functional component. Each component has 10 questions and is scored from 0–100 (min–max). The total score is divided by the number of questions answered giving an average score from 0–100. The tool was designed to be specific to adults with strabismus. Adults with other eye conditions and with normal eyes were included in its formation to achieve this specificity.

The overall scores were statistically analysed using the Wilcoxon Sign-rank Test, whilst the comparative subgroup analysis was performed using a two-tailed Mann Whitney U Test with confidence intervals (CI) set to 95%.

## Results

Pre and postoperative questionnaires of 35 patients were collected. Twenty females and 15 males were recruited. Sixteen patients had primary strabismus and 19 had recurrent strabismus (Table 1).

**Table 1:** Patient Demographics.

Total	35
Females	20
Males	15
Average Age	43.19
Esotropia	12
Exotropia	23
Primary	16
Recurrent	19

Overall pre and postoperative mean QOL scores are presented in Table 2.

**Table 2:** AS-20 Mean Quality of Life Scores in Patients Preoperatively and Postoperatively.

	AS–20 Score		
Overall	Average	Functional	Psychosocial

Preoperative	52.84 (CI 46.81 to 58.85)	62.38 (CI 55.82 to 68.95)	42.73 (CI 36.06 to 50.49)
Postoperative	67.12 (CI 60.50 to 73.60)	67.20 (CI 59.72 to 74.38)	67.05 (CI 59.88 to 74.22)

An overall average increase in quality of life of 14.29 (p = 0.0018) was demonstrated in all patients. The majority of this increase was in the psychosocial component of the questionnaire. There was no difference in mean change in QOL score between esotropias and exotropias [11.72 (CI –8.65 to 32.08) vs. 20.19 (CI 11.79 to 32.1); p = 0.3] or between primary and recurrent strabismus [16.55 (CI 8.27 to 27.95) vs. 18.11 (CI 5.83 to 27.27); p = 0.4]. Females did have statistically significantly lower preoperative mean QOL scores than males [46.78 (CI 39.36 to 54.21) vs. 60.89 (CI 52.26 to 69.53); p = 0.047] (Table 3). Females showed larger increases in mean QOL scores postoperatively but this did not reach statistical significance [21.05 (CI 12.4 to 29.7) vs. 5.12 (CI –7.61 to 17.85); p = 0.1].

**Table 3:** Comparison of Preoperative and Postoperative Mean AS-20 Quality of Life Scores in Males and Females.

	Males	Females	Difference (Males – Females)

Pre Avg	60.89 (CI 52.26 to 69.53)	46.78 (CI 39.35 to 54.21)	14.11
Pre Psych	53.82 (CI 44.21 to 63.43)	35.37 (CI 26.28 to 44.46)	18.45
Pre Func	67.96 (CI 58.09 to 77.84)	58.20 (CI 49.66 to 66.73)	9.76
Post Avg	66.01 (CI 55.69 to 76.33)	67.83 (CI 59.16 to 76.50)	–1.82
Post Psych	68.16 (CI 57.90 to 78.42)	66.22 (CI 56.10 to 76.34)	1.94
Post Func	63.86 (CI 50.90 to 76.82)	69.45 (CI 60.91 to 77.98)	–5.59
Diff Avg	5.12 (CI –7.60 to 17.84)	21.05 (CI 11.95 to 30.14)	–15.93
Diff Psych	14.34 (CI 0.56 to 28.11)	30.85 (CI 19.15 to 42.54)	–16.51
Diff Func	–4.1 (CI –17.56 to 9.36)	11.25 (CI 2.82 to 19.67)	–15.35

Avg = average, Psych = pyschosocial, Func = functional, Pre = preoperative, Post = postoperative.

Patients with lower mean preoperative QOL scores had greater changes in scores postoperatively. We found patients with high mean preoperative scores (67–100) to have a small net reduction in QOL score as shown in Table 4. There was a significant difference in mean QOL score change in the high group (66–100) compared with the moderate group (P = 0.01). There was also a significant difference between the high and the low group (P = 0.02) (Table 4). However, the high group maintained greater QOL scores postoperatively.

**Table 4:** AS-20 Mean Quality of Life (QOL) Score By Subgroup.

	Preoperative Mean QOL Score		
	Low (0–33)	Mod (34–66)	High (67–100)

Pre Avg	24.50 (CI 25.29 to 34.70)	50.21 (CI 50.59 to 57.26)	77.75 (CI 74.44 to 83.98)
Post Avg	61.00 (CI 50.21 to 76.73)	66.17 (CI 56.30 to 75.61)	73.82 (64.40 to 84.88)
Difference	36.50	15.96	–4.47

Avg = average, Pre = preoperative, Post = postoperative.

The average preoperative deviation was 36.8 prism diopters (PD) (CI 30.64 to 43.00) and the average postoperative deviation was 8.8PD (CI 6.28 to 11.36). In the patients who reported reductions in postoperative QOL scores, the average preoperative deviation was 36.2PD (CI 26.82 to 45.75) and the postoperative deviation was 8.6PD (CI 0.75 to 16.57). We found that there was a positive correlation between low preoperative QOL score and large deviation (R = 0.179) and a weak positive correlation between change in deviation and the change in QOL score (R = 0.086). However, complete postoperative orthoptic data was not attainable for 10 of the 35 patients and so the above results need to be interpreted with caution.

## Discussion

The main function of the AS-20 questionnaire is as a measurement tool. It can help to demonstrate the impact strabismus has on QOL preoperatively and can also help to measure subjective surgical outcomes postoperatively (Glasman et al. 2013). Clinicians sometimes struggle to assess the less tangible factors affecting a patient’s interpretation of surgical success. The AS-20 questionnaire allows us to quantify some of these factors.

Our analysis demonstrates a significant improvement in the quality of life following adult strabismus surgery. This has been reported in other studies and we hope lends further weight to the role of surgery in adult strabismus (Burke, Leach and Davis 1997; Jackson et al. 2006; Glasman et al. 2013). Beauchamp et al have previously shown that this benefit represents excellent healthcare economic value (2006). There was a significant difference in preoperative average scores between females and males (46.79 vs. 60.90; p = .047) which has been previously reported (Durnian et al. 2010). This difference was reduced following surgery with both males and females achieving similar mean postoperative average scores (67.83 vs. 66.01; p = 0.86). There was no difference in mean QOL score change between primary and recurrent strabismus cases. An interesting subgroup of the strabismus population is those in whom good surgical alignment and clinical results are achieved but postoperative QOL scores show a decrease. Some of these patients may have unrealistic expectations about the potential impact of surgery. Whilst surgery can help, it must be stressed that self-esteem, anxiety and other issues patients may have are extremely complex and often multifactorial.

In our subgroup analysis, we found a higher proportion of these patients came from our high (67–100) preoperative score group at 50% (4 out of 8) compared with 19% (4 out of 21) and 22% (1 out of 6) in the low and medium groups respectively. Whilst a reduction in QOL score does not necessarily equate with an unhappy patient or a patient regretting having the procedure performed, it could be interpreted as a degree of dissatisfaction.

A high preoperative score should encourage a more engaged discussion around patient expectations in relation to their QOL pre and postoperatively. By utilising the AS-20 score in this manner we do not mean to suggest that patients with higher scores should be advised against surgery. Quality of life scores from the normal population range from 95 to 84 (Hatt et al. 2009; Glasman et al. 2013). The average score in our high group was 77.5 which is still significantly inferior to the normal population. Patients in this group however, are less likely to register an increase and more likely to register a decrease in their QOL score. If the patient is unsure, further time to consider the procedure should be encouraged. This can help to set appropriate patient expectations and may aid candidate selection in equivocal cases.

A potential limitation of our audit was the exclusion of two patients undergoing revision surgery following a recent strabismus operation. Some strabismus cases are very complex and operations are undertaken in the knowledge that the strabismus is unlikely to be fully corrected. The two cases we excluded were cases of complex neurologic strabismus which we did not feel were representative of the wider adult strabismus population. Some of our cases had a relatively short follow up time, and it has previously been shown that QOL scores tend to improve over time postoperatively (Hatt et al. 2009; Glasman et al. 2013). Some of our patients with negative score changes may have increased over time. This evaluation was only concerned with the AS-20 scores. We did not have specific information in regards to patient satisfaction with the procedure. Finally, we must also acknowledge that the patients in our clinic actively sought care and may not be representative of the strabismus population as a whole.

## Conclusion

In the current climate of shrinking healthcare budgets and cutbacks it is important to collect and collate data regarding strabismus patients and their quality of life. This data will be critical in advocating on their behalf as further pressure is placed on adult strabismus surgery. This service analysis proves the effectiveness of adult strabismus surgery in improving quality of life in Irish patients. We also believe the AS-20 questionnaire may be used as a tool to help to reduce the number of poor subjective surgical outcomes associated with good objective surgical outcomes. We believe that greater consideration and care should be taken in operating on patients with high preoperative QOL. Our service evaluation demonstrates that these patients may be at higher risk of disappointing subjective outcomes after good clinical results but with detailed and careful discussion about expectations, this can potentially be minimized.

## Research Institution

This research was carried out in University Hospital Waterford, Ireland.
